# Subcellular localization and interactions of Infectious Salmon Anemia Virus (ISAV) M1 and NEP as well as host Hsc70

**DOI:** 10.1186/s12985-017-0702-z

**Published:** 2017-02-15

**Authors:** Wenting Zhang, Chengzhi Cai, Li Lin, Yizhi Jane Tao, Meilin Jin

**Affiliations:** 1 State Key Laboratory of Agricultural Microbiology, College of Veterinary Medicine, Huazhong Agricultural University, Wuhan, 430070 People’s Republic of China; 2 Department of Aquatic Animal Medicine, College of Fisheries, Huazhong Agricultural University, Wuhan, Hubei 430070 China; 3 Department of Biosciences, Rice University, Houston, TX USA

**Keywords:** Infectious salmon anemia virus, Subcellular localization, Protein interaction, Matrix protein, Nuclear export protein, Heat shock cognate 70

## Abstract

**Background:**

Infectious salmon anemia virus (ISAV) is an important fish pathogen that causes high mortality in farmed Atlantic salmon*.* The ISAV genome consists of eight single-stranded, negative-sense RNA segments. The six largest segments contain one open reading frame (ORF) each, and encode three polymerase proteins, nucleoprotein, fusion protein, and hemagglutinin esterase protein. The two smallest segments contain more than one ORF each. The segment 7 encodes non-structural protein 1 (NS1) and nuclear export protein (NEP), while segment 8 encodes matrix protein 1 and 2 (M1 and M2). NS1 and M2 have been well known as antagonist of type I interferon. However, little is known about the characterization of M1 or NEP. In addition, heat shock cognate 70 (Hsc70) has been reported to interact with M1 and NEP of influenza viruses for the export of viral ribonucleoprotein (vRNP) via vRNP-M1-NEP complex, the goal of this study therefore was to characterize the subcellular localization and interactions of ISAV M1 and NEP as well as cellular Hsc70.

**Results:**

When M1, NEP, and Hsc70 were individually expressed in the stripped snakehead (SSN-1) cells, we found that M1 protein was localized in both cytosol and nucleus of the cells, NEP was localized only in the cytosol and accumulated adjacent to the nucleus, while Hsc70 was localized throughout the cytosol, but not in the nucleus. However, when two of them were co-expressed, we found that both M1 and Hsc70 were co-localized with NEP in the cytosol and accumulated adjacent to the nucleus, while M1 and Hsc70 were still localized as they were expressed individually. Furthermore, pull-down assay was performed and showed that NEP could interact with both M1 and Hsc70, and M1-Hsc70 interaction was also observed although the interaction was weaker than that of NEP-Hsc70.

**Conclusion:**

Our study characterized the subcellular localization and interactions of three proteins including M1 and NEP of ISAV, and Hsc70. These data will help towards a better understanding of the life cycle of ISAV, especially the process of vRNP export.

## Background

Viruses that belong to the family *Orthomyxoviridae* consist of segmented, single-stranded, and negative-sense RNAs. Based on their genetic characterization and host range, the members of the *Orthomyxoviridae* family are divided into five genera including influenza A, B, and C viruses, Thogotovirus, and Isavirus [1]. The infectious salmon anemia virus (ISAV) is the only species in the genus Isavirus [1]. Its infection has caused serious diseases in farmed Atlantic salmon (*Salmo salar*) in Norway, Canada, the United States, Scotland, and Chile [1–4].

In common with influenza A and B viruses, ISAV is also composed of eight RNA segments [5]. The six largest segments contain one open reading frame (ORF) each, while the two smallest segments contain more than one ORF each. The segments 1–4 encode three polymerase proteins and nucleoprotein, which bind with viral RNAs to form viral ribonucleoproteins (vRNPs) [6]. Segments 5 and 6 encode two surface proteins: fusion protein and hemagglutinin esterase protein [7–9]. Segment 7 exhibits similar coding strategy with the segment 7 of influenza A and B viruses, resulting in a linear ORF1 and a spliced ORF2 [10]. These two ORFs encode a non-structural protein 1 (NS1) and a nuclear export protein (NEP), which are different from the segment 7 of influenza viruses that encode matrix protein 1 and 2 (M1 and M2) [11]. Segment 8 of ISAV contains two linear ORFs encoding two proteins with 196 and 241 amino acids [1]. The larger protein encoded by ORF2 is M2 protein, while the smaller one encoded by ORF1 is the M1 protein [11, 12]. The NS1 and M2 proteins of ISAV are antagonists of type I interferon [13, 14]. However, the characterization of M1 or NEP was still unclear.

Hsc70, a constitutive form of Hsp70 family protein, is involved in cell entry of rotavirus [15], and mediates viral RNP export of influenza virus by interacting with M1-NEP complex [16, 17]. However, whether Hsc70 was involved in the life cycle of ISAV was unknown. In this study, the subcellular localization of ISAV M1 and NEP, as well as Hsc70 was investigated when they were expressed in SSN-1 cells. In addition, the interactions of the three proteins were performed using pull-down array. Our study provides data that will help further studies on ISAV M1 and NEP.

## Results and discussion

### Subcellular localization of ISAV M1 and NEP

In orthomyxoviruses, the segment 7 of influenza A and B viruses can generate a linear and a spliced transcript, which respectively encode M1 and M2 proteins [18, 19]. As the ISAV segment 7 also generates a linear and a spliced transcript with similar splicing strategy to the segment 7 of influenza viruses, the ISAV segment 7 was originally assumed to encode M1 and M2 proteins [20]. However, Kibenge et al. revealed that the two proteins encoded by the ISAV segment 7 were actually NS1 and NEP [21]. Instead, the ORF1 and ORF2 of ISAV segment 8 was confirmed to encode M1 and M2 proteins [12].

**Fig. 1:**
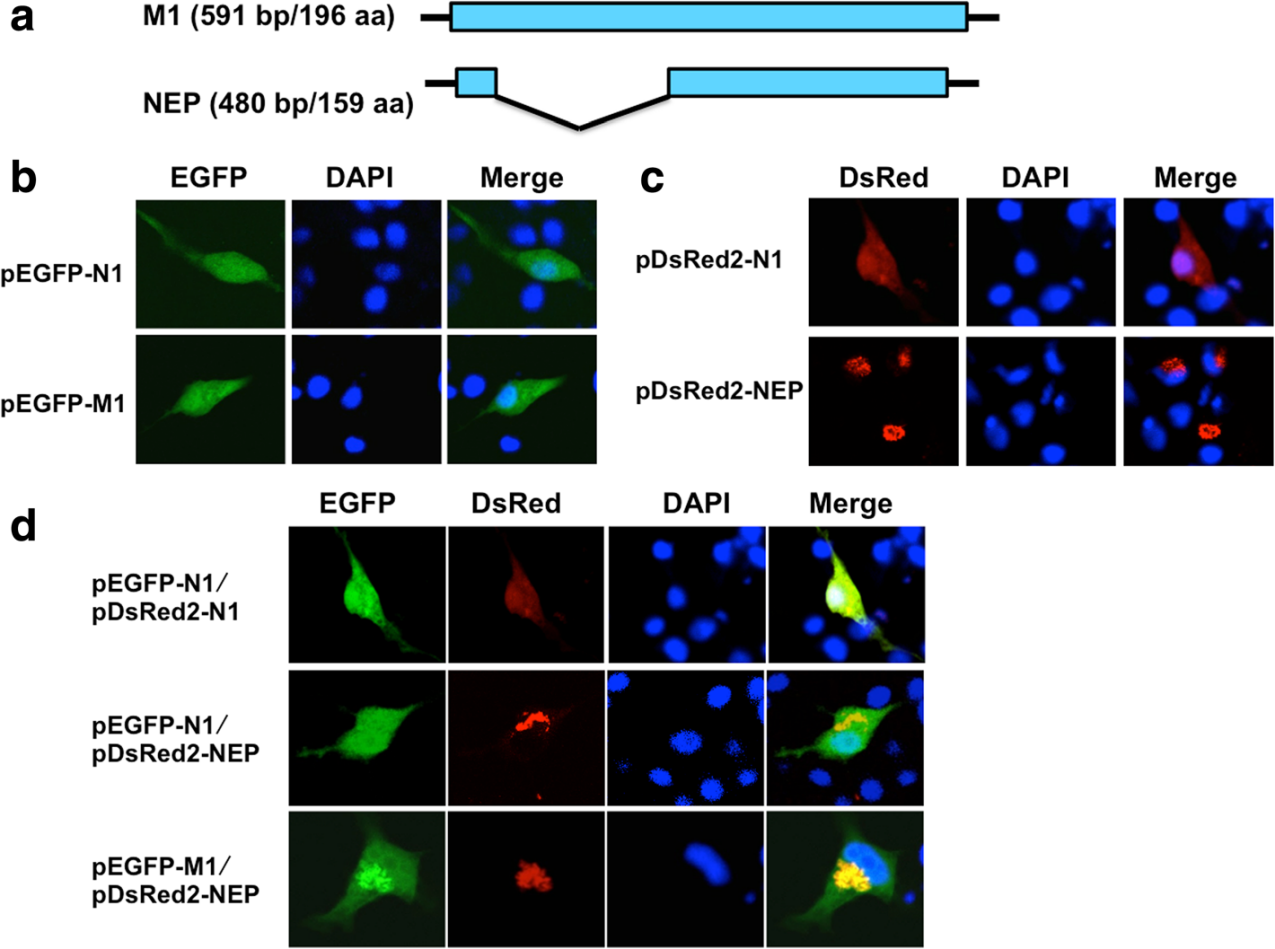
Co-localization of M1 and NEP in SSN-1 cells. **a** Schematic representation of M1 and NEP. **b**-**d** Subcellular localization of M1 or/and NEP in SSN-1 cells. SSN-1 cells were transfected with pEGFP-N1 or pEGFP-M1 (**b**), pDsRed2-N1 or pDsRed2-NEP (**c**), pEGFP-N1 and pDsRed2-N1, pEGFP-N1 and pDsRed2-NEP, or pEGFP-M1 and pDsRed2-NEP (**d**). At 24 h post-transfection, cells were fixed with 4% paraformaldehyde, permeabilized with 0.2% Triton X-100 and incubated DAPI. Cells were imaged on a LSM510 Meta confocal laser-scanning microscope

The ISAV M1 protein is 196 amino acids (aa) in length (Fig. 1a). To determine the subcellular localization of ISAV M1 protein, we constructed a plasmid pEGFP-M1, which expressed enhanced green fluorescent protein (EGFP)-tagged M1. In plasmid pEGFP-M1 or the empty vector pEGFP-N1 transfected SSN-1 cells (Fig. 1b), we found that EGFP-M1 showed green fluorescence in both cytosol and nucleus of the SSN-1 cells (Fig. 1b), similar to that observed in pEGFP-N1 transfected SSN-1 cells.

The ISAV NEP is encoded by a spliced ORF with 159-aa in length (Fig. 1a). To investigate the subcellular localization of NEP, the ISAV NEP gene was cloned into pDsRed2-N1 vector to generate plasmid pDsRed2-NEP, which expressed discosoma red fluorescent protein (DsRed)-tagged NEP. In transfected SSN-1 cells, the fluorescence protein DsRed itself showed red fluorescence throughout the SSN-1 cells, while the DsRed-NEP was localized only in the cytosol and accumulated adjacent to the nucleus of SSN-1 cells (Fig. 1c). Previous study have revealed that the ISAV NEP accumulated around the nucleus in plasmid pCDNA-Flag-NEP transfected Epithelioma papulosum cyprinid (EPC) cells [10]. Our results were consistent with the localization of NEP in EPC cells [10], indicating the accumulation of NEP around the nucleus was a common feature.

To illuminate the subcellular localization of M1 and NEP when they were co-expressed, the plasmids pEGFP-M1 and pDsRed2-NEP was co-transfected into SSN-1 cells (Fig. 1d). We found that M1 was co-localized with NEP in cytosol and accumulated adjacent to the nucleus (Fig. 1d). As control, in transfected SSN-1 cells, we found that EGFP itself did not co-localize with DsRed-NEP or DsRed (Fig. 1d). The results suggested that M1 probably interacted with NEP in SSN-1 cells.

### Interaction between ISAV M1 and NEP

**Fig. 2:**
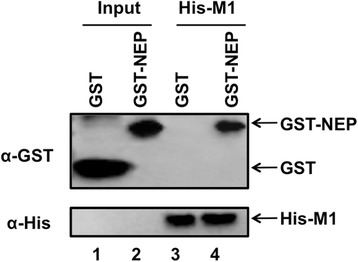
Interaction between M1 and NEP protein detected by pull-down assay. The His-M1 protein (10 μg) was incubated with 10 μg of GST or GST-NEP. To detect His-M1 and GST-NEP, western blot analysis was performed using either anti-His (lower panel) or anti-GST (upper panel) antibodies. In this assay, 1/100 of the total volume of His-M1 protein was loaded as the input (lanes 1 and 2), and 1/10 of the total volume of each eluted sample was loaded to detect interacted proteins (lanes 3 and 4)

To further confirm the interaction between ISAV M1 and NEP, we generated plasmids expressing His-M1 or GST-NEP. These proteins were prokaryotically expressed and purified. Pull-down array showed that His-M1 could interact with GST-NEP, but not with GST. These data indicated that ISAV M1 could interact with NEP (Fig. 2).

### Determination of the co-localization of Hsc70 with ISAV M1 or/and NEP

**Fig. 3:**
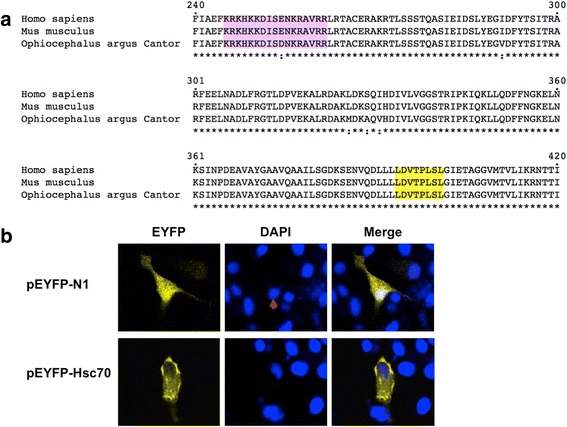
Subcellular localization of Hsc70 protein in SSN-1 cells. **a** Sequence comparison of Hsc70 originated from human, mouse, and snakehead fish. CLUSTALW was used to align the amino acid sequences of Hsc70 (240-420 aa) from human (Homo sapiens), mouse (Mus musculus), and snakehead fish (Ophiocephalus argus Cantor). The nuclear localization signals (*purple*) and the nuclear export signals (*yellow*) are highlighted. **b** SSN-1 cells were transfected with the plasmid pEYFP-N1 or pEYFP–Hsc70. At 24 h post-transfection, cells were fixed with 4% paraformaldehyde, permeabilized with 0.2% Triton X-100 and incubated DAPI. Cells were imaged on a LSM510 Meta confocal laser-scanning microscope

In addition to M1-NEP interaction that is essential for viral RNPs export, some other host factors such as Hsc70 has also been reported to facilitate viral RNPs export of influenza viruses [16, 17, 22]. Hsc70, a constitutive form of Hsp70 family, has been reported to be involved in the propagation of several viruses [23, 24]. Hsc70 contains a nuclear localization signal (NLS) and a NES (Fig. 3a) [25, 26], and was previously reported to bind with M1 protein of influenza A virus to facilitate RNP complex export from nucleus to cytosol [17]. In this study, the Hsc70 gene was amplified from SSN-1 cells and cloned into vector pEYFP-N1 to generate a plasmid pEYFP-Hsc70, which expressed enhanced yellow fluorescent protein (EYFP)-tagged Hsc70. In transfected SSN-1 cells, EYFP itself showed yellow fluorescence in both cytosol and nucleus of SSN-1 cells, while EYFP-Hsc70 showed fluorescence only in cytosol, the protein seemed to be excluded from the nucleus (Fig. 3b).

**Fig. 4:**
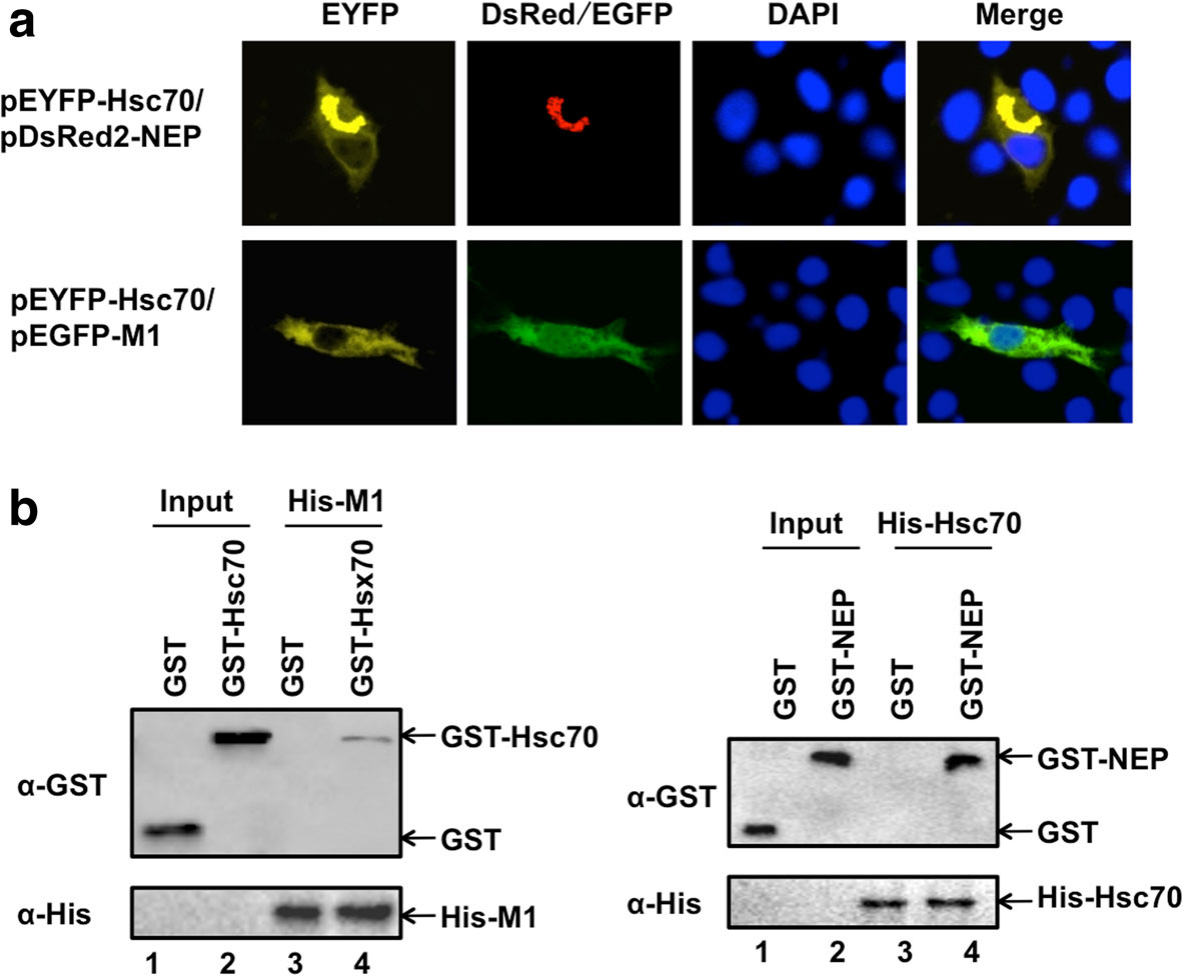
Co-localization and interaction of Hsc70-M1 with M1 or/and NEP. **a** Co-localization of Hsc70-M1 and Hsc70-NEP. SSN-1 cells were transfected with pEYFP-Hsc70 and pDsRed2-NEP, or pEYFP-Hsc70 and pEGFP-M1. At 24 h post-transfection, cells were fixed with 4% paraformaldehyde, permeabilized with 0.2% Triton X-100 and incubated DAPI. Cells were imaged on a LSM510 Meta confocal laser-scanning microscope. **b** Interaction of Hsc70-M1 and Hsc70-NEP. The His-M1 protein (10 μg) or His-Hsc70 (10 μg) was incubated with 10 μg GST, GST-Hsc70 or GST-NEP. Western blot analysis was performed using either anti-His (lower panel) or anti-GST (upper panel). In this assay, 1/100 of the total volume of His-M1 or His-Hsc70 was loaded as the input (lanes 1 and 2) and 1/10 of the total volume of each eluted sample was loaded to detect interacted proteins (lanes 3 and 4)

To determine the possible co-localization of Hsc70 with M1 or/and NEP, the plasmid pEYFP-Hsc70 was co-transfected with pEGFP-M1 or pDsRed2-NEP. The results showed that Hsc70 and M1 were still localized as they were expressed individually. However, Hsc70 was observed to co-localize with NEP in the cytosol and accumulated adjacent to the nucleus of SSN-1 cells (Fig. 4a). The results indicated that Hsc70 probably interacted with NEP, but whether it interacted with M1 needed to be determined further.

### Interaction between Hsc70 and ISAV M1 or/and NEP

To investigate the interactions of Hsc70 with ISAV M1 or/and NEP, we generated two plasmids expressing GST-Hsc70 or His-Hsc70 respectively (Fig. 4b). GST-Hsc70 was used to study the interaction with His-M1, while His-Hsc70 was used to study the interaction with GST-NEP. Pull-down array showed that a weak band of GST-Hsc70 was pulled down by His-M1, indicating that Hsc70 could interact with M1 (Fig. 4b). It has been previously reported that Hsc70 could interact with M1 protein of influenza virus [17], indicating that Hsc70-M1 interaction might play a similar role in the life cycle of ISAV and influenza viruses. In addition, a band of GST-NEP, but not GST alone, was pulled down by His-Hsc70, indicating that Hsc70 could also interact with NEP (Fig. 4b). Therefore, it is hypothesized that the M1 and NEP of ISAV, and Hsc70 interact with each other to form a complex, which may play important roles in the export of vRNP of ISAV.

**Fig. 5:**
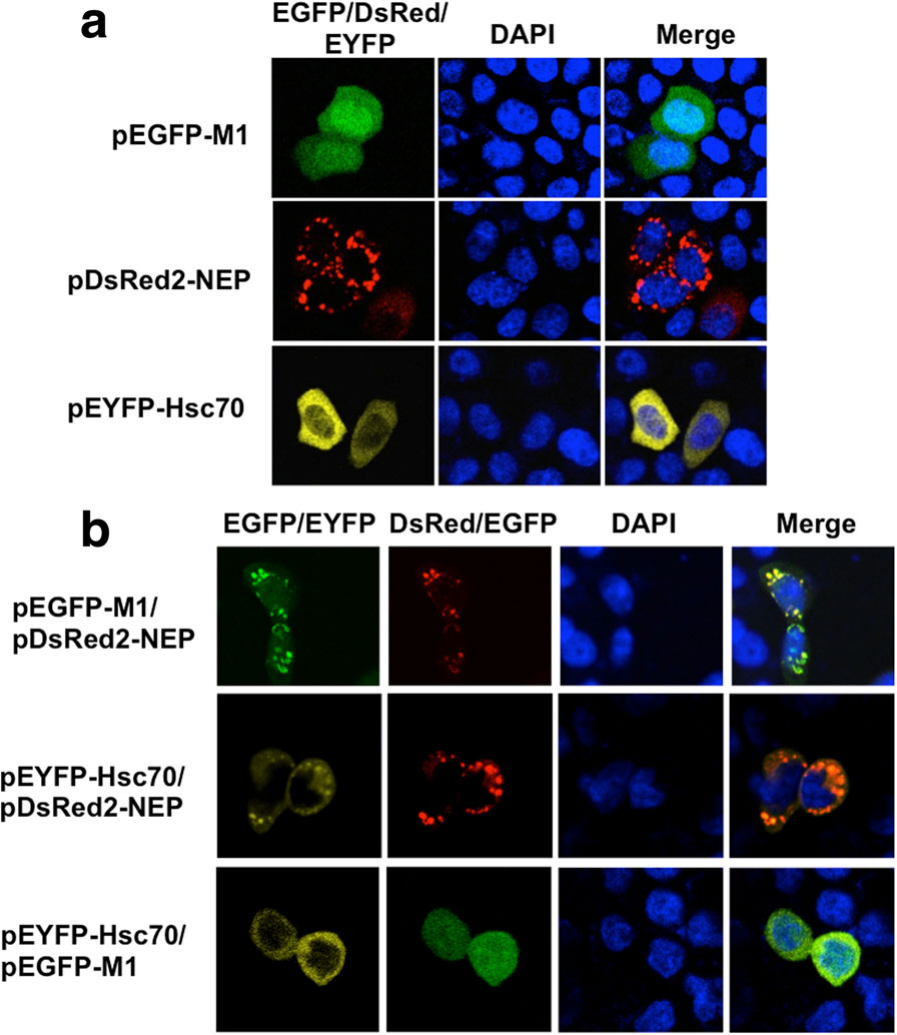
Co-localization between M1 and NEP, Hsc70 and NEP, Hsc70 and M1 in Hela cells. Hela cells were transfected with pEGFP-M1, pDsRed2-NEP or pEYFP-Hsc70 (**a**), plasmids pEGFP-M1 and pDsRed2-NEP, pEYFP-Hsc70 and pDsRed2-NEP, or pEYFP-Hsc70 and pEGFP-M1 (**b**). At 24 h post-transfection, cells were fixed with 4% paraformaldehyde, permeabilized with 0.2% Triton X-100 and incubated DAPI. Cells were imaged on a LSM510 Meta confocal laser-scanning microscope

In order to exclude non-specific effects caused by cell origin, the subcellular localization of each protein as well as the co-localization of the proteins were investigated in human-origin Hela cells (Fig. 5a). Consistent with the observation in SSN-1 cells, the EGFP-M1 was localized throughout the cells, DsRed-NEP was localized in the cytosol and accumulated around the nucleus, while the Hsc70 was exclusively localized in the cytosol. Furthermore, Co-localization could also be observed between M1 and NEP as well as Hsc70 and NEP (Fig. 5b), which were consistent with the observations in SSN-1 cells.

## Conclusion

In this study, the subcellular localization and interactions of M1 and NEP of ISAV, and Hsc70 was investigated. We found that the three proteins can interact with each other. However, although several protein-protein interactions have been illuminated, further work is still needed to illuminate whether these interactions have any effects on ISAV propagation.

## Methods

### Plasmids and antibodies

The plasmid pET28b-M1, which expresses His-M1, was kindly provided by Dr. Yizhi Jane Tao from the department of biosciences, Rice University. The plasmid pGEX2T-NEP, expressing GST-NEP, was constructed by synthesizing the NEP gene of ISAV (accession number: EF523765.1) and cloning into the pGEX2T. The plasmids pET28b-Hsc70 and pGEX2T-Hsc70, expressing His-Hsc70 or GST-Hsc70, were constructed by ligating the reverse transcription polymerase chain reaction (RT-PCR) amplified product of Hsc70 from the RNA extracted from SSN-1 cells into pET28b or pGEX2T, respectively. The plasmids pEGFP-M1, pDsRed2-NEP and pEYFP-Hsc70 were constructed by ligating the PCR-amplified products into the plasmid pEGFP-N1, pDsRed2-N1 or pEYFP-N1, with primers as listed in Table 1. The anti-GST and anti-His antibodies were purchased from ABclonal and PML Biotechnology co, respectively.

**Table 1:** Primers used in this study

Plasmid	Primer	Sequences
pEGFP-M1	pEGFP-M1-F	CCGCTCGAGATGAACGAATCACAATGGATACAA
	pEGFP-M1-R	CGCGGATCCCGCTTCAGGTACCCCAGAAGCAC
pDsRed2-NEP	pDsRed2-NEP-F	CCGCTCGAGATGGATTTCACCAAAGTGTA
	pDsRed2-NEP-R	CCGGAATTCGGTTCTCATTACAAATGAATT
pEYFP-Hsc70	pEYFP-Hsc70-F	CCGCTCGAGATGTCTAAGGGACCAGCAGTTG
	pEYFP-Hsc70-R	CGCGGATCCCGATCAACCTCCTCGATGGTGGGA

### Expression and purification

The expression of His-M1, His-Hsc70, GST-Hsc70, and GST-NEP were induced with 1 mM IPTG at 15 °C for 20 h after the cell density reached an OD_600_ nm of 0.6. Cells were collected and sonicated in a lysis buffer [300 mM NaCl, 5 mM Imidazole, 10% (v/v) glycerol, and 50 mM Tris · HCl (pH 7.5)]. The lysate was centrifuged at 12,000 rpm for 40 min, and the supernatant was used for further purification. The His-tagged proteins were purified using HisPur Ni-NTA Resin (GE Healthcare), while GST-tagged proteins were purified using glutathione sepharose 4B manual (GE Healthcare). All purified proteins were dialyzed in a NET/NP-40 buffer [50 mM Tris · HCl (pH 7.9), 0.1 M NaCl, 5 mM EDTA, and 0.1% NP-40], which was also used as wash buffer.

### Pull-down assays

To determine protein-protein interactions, the His-tagged proteins were respectively bound to 50 μl HisPur Ni-NTA Resin (GE Healthcare) and then incubated with GST-tagged proteins at 4 °C for 1 h. After washed three times with the NET/NP-40 buffer at 4 °C, the beads were eluted with 250 mM Imidazole and then subjected to western blot analysis using both anti-GST and anti-His antibodies.

### Transfection

To observe the subcellular localization of M1, NEP, and Hsc70, as well as possible co-localization between these proteins, SSN-1 or HeLa cells in 24-well plates were transfected with 2.0 μg of plasmid pEGFP-N1, pDsRed2-N1, pEYFP-N1, pEGFP-M1, pDsRed-NEP, pEYFP-Hsc70, or a series of combinations using FuGENE HD (Roche, WI). Fresh medium was replaced at 6 h post transfection. At 24 h post transfection, the cells were fixed with 4% paraformaldehyde, permeabilized with 0.2% Triton X-100 and incubated for 10 min with 4’,6- diamidino-2-phenylindole (DAPI). Cells were imaged on a LSM510 Meta confocal laser-scanning microscope.

## Abbreviations

DAPI: 4’,6- diamidino-2-phenylindole
DsRed: Discosoma red fluorescent protein
EGFP: Enhanced green fluorescent protein
EPC: Epithelioma papulosum cyprinid
EYFP: Enhanced yellow fluorescent protein
Hsc70: Heat shock cognate 70
ISAV: Infectious salmon anemia virus
M1: Matrix protein 1
M2: Matrix protein 2
NEP: Nuclear export protein
NES: Nuclear export signal
NLS: Nuclear localization signal
NS1: Non-structural protein 1
ORF: Open reading frame
RT-PCR: Reverse transcription polymerase chain reaction
SSN-1: Stripped snakehead
vRNP: Viral ribonucleoprotein
